# South West Orthopaedic Club Abstracts of Meeting in Exeter, November 1984

**Published:** 1985-04

**Authors:** 


### South West Orthopaedic Club

Meeting at Exeter on 3rd November 1984

### ACETABULAR BONE DESTRUCTION IN OSTEOARTHRITIS RELATED TO ANTI-INFLAMMATORY MEDICATION

**N. M. Newman, Exeter**

Indomethacin and other Non-Steroidal Anti-Inflammatory Drugs (NSAID) have been shown to have a deleterious effect on bone repair and remodelling.

In the osteoarthritic hip accelerated destruction and femoral head necrosis have been associated with NSAID use.

Acetabular destruction can significantly increase the difficulty of performing hip replacement and is, therefore, a much more important consideration than femoral head necrosis. In this study acetabular migration was used as a measure of acetabular destruction. Fifty-eight patients undergoing total hip replacement at the Princess Elizabeth Orthopaedic Hospital for primary osteoarthritis were studied. Thirty-six patients undergoing 38 hip replacements had adequate sequential X-rays and drug history available.

Twenty-three hips showed significant acetabular migration and all but one of these hips were in patients taking regular NSAID. Of the 15 hips not showing migration only 3 were in patients taking regular NSAID. The two groups of patients did not differ significantly in age, sex or femoral valgus angles. Thus, a significant correlation between regular ingestion of NSAID and acetabular bone destruction was found. This finding adds evidence to the association of NSAID with impaired bone remodelling and suggests that their use may be harmful, both before and after hip replacement.

### INDOMETHACIN IN THE PREVENTION OF ECTOPIC BONE FORMATION AFTER TOTAL HIP REPLACEMENT

**T. D. Bunker, R. L. M. Newell and R. S. M. Ling, Exeter**

Ectopic ossification occurs in 10–15% of patients following total hip replacement, and is symptomatic in 5%. Symptoms occur at 12–21 days and consist of pain, tenderness and low grade pyrexia. Ectopic ossification is common in patients with Forrestiers disease and occurs in 92% of patients who have formed ectopic bone following a previous total hip replacement. Diphosphonates are of little use but radiotherapy (2000 rad) or Indomethacin have been shown to reduce ectopic bone formation considerably.

This study presented three patients who had each had one of their hips previously replaced and had formed ectopic bone around the prosthesis. When they came to have the other hip replaced they were given Indomethacin, 25 mg orally three times daily for three weeks. Despite a 92% chance of ectopic bone formation, two formed no bone around the second prosthesis and one a minimal amount. This result is highly significant and we would suggest that all patients at high risk of ectopic bone formation are given prophylactic Indomethacin for three weeks following operation.

### THE RADIOLOGICAL APPEARANCE OF DIFFERENT TYPES OF ACETABULAR CYST WITH PARTICULAR REFERENCE TO CHONDROSARCOMA OF THE PELVIS

**A. G. MacEachern G. H. Heyse-Moore, Exeter**

The radiological appearance of the lytic type of central chondrosarcoma has not been well described in the literature. Six patients with chondrosarcoma of the pelvis were described all of whom presented with hip pain and had an ill-defined cystic appearance of the subchondral bone of the acetabular roof as the presenting radiological finding. This appearance was misdiagnosed in all six cases by experienced orthopaedic surgeons and radiologists.

The differential diagnosis of cysts in the acetabular roof was discussed and the particular features suggestive of early chondrosarcoma were described.

### THE MANAGEMENT OF CURLY TOES IN CHILDREN BY FLEXOR TO EXTENSOR TRANSFER

**J. M. Digby, Cardiff and Mr. D. A. Jones, Swansea**

Recommended treatment for congenital curly toes varies from one surgeon to another. A retrospective review of children treated with flexor to extensor transfer was therefore performed.

All children treated in Swansea between 1964 and 1978 were called for review. Thirty-nine (83%) were reviewed; 33 attended for examination, and 6 replied to a postal questionnaire. The mean follow-up interval was 9 years (range 3–18 years). Twenty-eight of the patients examined had bilateral surgery and a total of 106 toes were treated.

The average age at operation was $7\frac{1}{2}$ years (range

4–15 years). Advice had been sought because of the appearance of the toes, discomfort whilst wearing shoes, or the presence of fixed flexion deformities in older members of the same family.

The operative procedure is well described. Difficulty isolating the long flexor tendon may be overcome by flexing and internally rotating the toe, which virtually dislocates the tendon into the wound once the flexor sheath is incised. In this series, the short flexor tendons were preserved.

We have noted that the long flexor tendon is almost bipartite in the lesser toes, and therefore it is important that the whole tendon is transferred. This anatomical finding has been confirmed by dissection of 20 adult cadaver lesser toes. Section of a third toe of a 22 week foetus show a fibroblast column separating the long flexor tendon, which indicates the development of the bipartite appearance.

The children reviewed had 35 good results, 2 fair and 2 poor. The patients with fair results had minor discomfort, and those with poor results had residual flexion deformities and discomfort. These deformities may have resulted from transfer of only part of the long flexor tendon.

These results are comparable with those of flexor tenotomy, which is a simpler operation. However, flexor to extensor transfer corrects rotational deformity, and preservation of the short flexor tendons retains mobility at the proximal inter-phalangeal joints.

### CLOSURE OF OSTEOMYELITIC AND TRAUMATIC DEFECTS OF THE LEG BY MUSCLE AND MUSCULOCUTANEOUS FLAPS

**E. T. R. James and J. S. Gruss, Bristol**

A retrospective review was carried out of 17 muscle and musculocutaneous flaps in the leg performed for traumatic and osteomyelitic defects in two groups of patients. One group of ten patients with soft-tissue defects resulting from trauma underwent muscle flap coverage in nine and musculocutaneous flap cover in one. Nine of the ten healed uneventfully. The second group of seven patients with defects resulting from chronic osteomyelitis underwent muscle flap cover in two, musculocutaneous flap cover in three, and combined muscle and musculocutaneous flap cover in two. All seven of the flaps transposed healed well without recurrence of infection. This series illustrates that the technique of muscle and musculocutaneous flap transposition can provide a safe, relatively straightforward method of cover for defects of the leg from trauma and chronic osteomyelitis with predictable results.

